# A novel method to efficiently differentiate human osteoclasts from blood-derived monocytes

**DOI:** 10.1186/s12575-024-00233-6

**Published:** 2024-03-19

**Authors:** Suganja Chandrabalan, Linh Dang, Uwe Hansen, Melanie Timmen, Corinna Wehmeyer, Richard Stange, Tim Beißbarth, Claudia Binder, Annalen Bleckmann, Kerstin Menck

**Affiliations:** 1 Department of Medicine A, Hematology, Oncology, and Pneumology, University of Muenster, Muenster, Germany; 2 West German Cancer Center, University Hospital Muenster, Muenster, Germany; 3 Department of Medical Bioinformatics, University Medical Center Goettingen, Goettingen, Germany; 4 Institute of Musculoskeletal Medicine (IMM), University of Muenster, Muenster, Germany; 5 Department of Hematology/Medical Oncology, University Medical Center Goettingen, Goettingen, Germany

**Keywords:** Osteoclasts, Monocytes, Macrophages, Teflon bags, Osteoclastogenesis

## Abstract

**Background:**

Osteoclasts are the tissue-specific macrophage population of the bone and unique in their bone-resorbing activity. Hence, they are fundamental for bone physiology in health and disease. However, efficient protocols for the isolation and study of primary human osteoclasts are scarce. In this study, we aimed to establish a protocol, which enables the efficient differentiation of functional human osteoclasts from monocytes.

**Results:**

Human monocytes were isolated through a double-density gradient from donor blood. Compared to standard differentiation schemes in polystyrene cell culture dishes, the yield of multinuclear osteoclasts was significantly increased upon initial differentiation of monocytes to macrophages in fluorinated ethylene propylene (FEP) Teflon bags. This initial differentiation phase was then followed by the development of terminal osteoclasts by addition of Receptor Activator of NF-κB Ligand (RANKL). High concentrations of RANKL and Macrophage colony-stimulating factor (M-CSF) as well as an intermediate cell density further supported efficient cell differentiation. The generated cells were highly positive for CD45, CD14 as well as the osteoclast markers CD51/*ITGAV* and Cathepsin K/*CTSK*, thus identifying them as osteoclasts. The bone resorption of the osteoclasts was significantly increased when the cells were differentiated from macrophages derived from Teflon bags compared to macrophages derived from conventional cell culture plates.

**Conclusion:**

Our study has established a novel protocol for the isolation of primary human osteoclasts that improves osteoclastogenesis in comparison to the conventionally used cultivation approach.

## Introduction

Bone is a rigid, yet dynamic organ. Throughout life, it is continuously remodeled, shaped and repaired. The remodeling process involves the breakdown (resorption) and build-up (synthesis) of bone mass [1]. Several resident cell populations are involved in shaping the bone, including osteocytes, osteoblasts and osteoclasts [2]. Among them, osteoclasts are unique as they are the main cell type that is able to resorb bone [3]. Therefore, osteoclasts play a pivotal role in bone homeostasis and bone damage under pathological conditions. Since excessive osteoclast formation causes bone loss in the most prevalent forms of osteoporosis, e.g. postmenopausal osteoporosis, or bone loss due to inflammation and bone metastasis, osteoclasts are the primary targets for many antiresorptive therapies [1]. Thus, in vitro cell culture models, which enable the cultivation of highly active osteoclasts, are required for studies evaluating the efficiency of bone resorption inhibitors and other clinically relevant analyses.

Osteoclasts arise from the monocyte-macrophage lineage. The cells need to undergo a complex multistep differentiation process [3], including the fusion of lineage-committed mononuclear precursors, for the generation of multinucleated osteoclasts. Studies have identified two hematopoietic factors that are crucial for osteoclastogenesis: macrophage colony-stimulating factor (M-CSF) and the receptor-activator of NF-κB Ligand (RANKL) [4–6]. M-CSF is required for the differentiation of hematopoietic stem cells (HSCs) into the monocyte/macrophage lineage, promoting proliferation and extension of the lifespan of precursor cells [7, 8]. However, for terminal osteoclast differentiation, RANKL is the critical factor. RANKL stimulates the pool of M-CSF-expanded precursors to commit to the osteoclast phenotype by inducing the differentiation, fusion and lifespan-expansion of osteoclasts [3]. In the field of bone research, it has been well established that these two factors are sufficient to induce osteoclastogenesis in vitro.

Based on these findings, different in vitro culture systems have evolved over time: the most widely-used osteoclast culture systems are either based on the murine macrophage cell line RAW264.7 [9], or on primary murine bone marrow cells [10]. However, isolation of the latter requires the sacrifice of mice. More importantly, as both cell types are from murine origin, the translatability to the human setting remains questionable [11]. Using osteoclast precursors of human origin would overcome these obstacles. Only few studies are available which use human-derived precursors for osteoclast differentiation. One possibility is to use the human monocytic cell line THP-1 for osteoclastogenesis [12]. However, these cells were isolated from a patient with acute leukemia, and as such, the monocytes have undergone malignant transformation and hence might not reflect the physiological behavior of healthy osteoclasts [13]. A more promising alternative is to isolate human peripheral blood mononuclear cells (PBMCs) as the basis for osteoclast differentiation and cultivation. Only few studies have used human PBMCs as osteoclast precursors, although they harbor the advantage of easy harvest from blood and enable a syngeneic in vitro culture model for studies in the human setting [13, 14].

Therefore, the aim of this study was to establish a culture method, which allows an efficient, cost-effective differentiation of human PBMCs to highly active osteoclasts. The method presented here enables investigators to use functionally active, mature osteoclasts for different downstream analyses, which have so far been limited due to the low number of obtained cells and the lack of possibilities for re-seeding of defined cell numbers for planned experiments.

## Materials and methods

### Isolation and differentiation of monocytes to osteoclasts

The blood for isolation of human monocytes from healthy donors was kindly provided by the Department of Transfusion Medicine, University Hospital Muenster. The monocytes were isolated via a double-density gradient centrifugation protocol as described previously [15]. As starting material, blood from either buffy coats or leukoreduction system chambers can be used. Briefly, 10 ml of donor blood were diluted with 20 ml PBS containing 1 mM EDTA. The suspension was layered carefully on top of 15 ml of a Ficoll gradient (Pancoll, PANbiotech) and was centrifuged at 400 × g for 45 min (without brake). The interphase between the plasma and Ficoll layer was collected with a plastic Pasteur pipet and washed twice with PBS containing 1 mM EDTA at 400 × g for 10 min (without brake). The PBMC pellet was resuspended in 20 ml RPMI-1640 medium without phenol red and carefully layered on top of 25 ml of a 46%-iso-osmotic Percoll gradient (Fisher Scientific). The iso-osmotic gradient contains 92% of Percoll solution in 10 × PBS (w/o Ca^2+^ and Mg^2+^) and 8% of RPMI-1640 + 10% heat-inactivated (56 °C, 30 min) fetal calf serum (FCS). The sample was centrifuged at 550 × g for 30 min (without brake). Again, the interphase was collected with a plastic Pasteur pipet and washed once in 50 ml PBS containing 1 mM EDTA by centrifugation at 400 × g for 10 min (without brake). The monocyte pellet was resuspended in 10 ml RPMI-1640 containing 10% FCS.

**Fig. 1:**
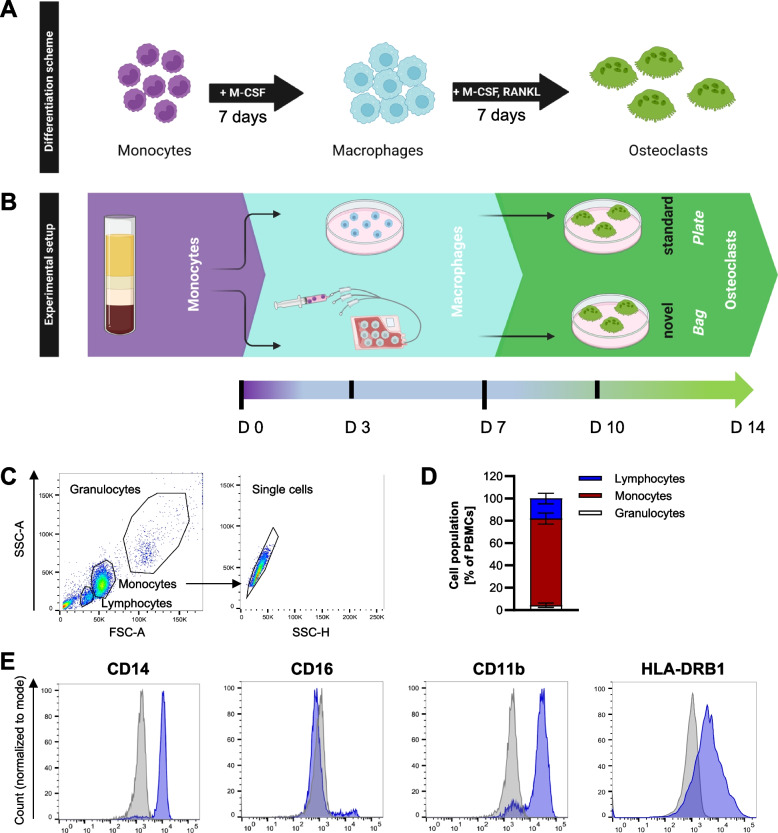
Differentiation of primary human osteoclasts. **A** Differentiation scheme of monocytes to osteoclasts. **B** Monocytes were isolated from peripheral blood and differentiated for 7 days to macrophages in the presence of M-CSF either in polystyrene cell culture plates (standard method) with a medium change every 3 days or in Teflon bags (novel method) with no medium change required. For terminal osteoclast differentiation, cells were either harvested from the bags and seeded at the desired cell density in cell culture plates, or kept adherent in plates. In both approaches, cells were cultured for an additional 7 days in a medium containing both M-CSF and RANKL which was changed every 3 days.** C** + **D**, Flow cytometry profile and gating strategy (**C**) including corresponding quantification (**D**) of the monocyte preparations. **E** Expression of surface marker proteins CD14, CD16, CD11b, CD163 and HLA-DRB1 was assessed on monocytes from n > 3 donors. Shown is one representative histogram for each marker. Blue: antibody-of-interest, grey: isotype control

The isolated monocytes were first differentiated to macrophages. To this end, monocytes were either differentiated in 32 ml VueLife cell culture bags coated with a thin fluorinated ethylene propylene (FEP) Teflon film (5 Mil (0.005’’ thick)) (CellGenix) or in regular cell culture dishes with a polystyrol plastic surface for adherent cells (Sarstedt). The Teflon bags have an inside surface area of 87 cm^2^ and allow regular gas exchange, but otherwise constitute a closed system environment. For differentiation of monocytes in Teflon bags, 2.5 × 10^7^ cells were seeded in 15 ml RPMI-1640 + 10% FCS, 2% human AB serum and M-CSF, while cells differentiated in cell culture plates were cultivated in RPMI-1640 + 10% FCS supplemented with M-CSF. Cells were differentiated to macrophages for 7 days at 37 °C and 5% CO_2_. During this differentiation period, the cells cultivated in Teflon bags remain in suspension, which facilitates the harvesting process which is described in detail in [15]. After harvesting the macrophages from the bags, they were seeded in cell culture dishes for further differentiation to osteoclasts, or remained in their initially seeded culture dishes. For osteoclast differentiation, cells were cultured in RPMI-1640 + 10% FCS supplemented with both M-CSF and RANKL for another 7 days, with change of culture medium every three days. The differentiation scheme is summarized in Fig. 1.

### Flow cytometry

To detach the macrophages/osteoclasts from the cell culture dishes, the plates were incubated on ice for 20 min and then washed once with PBS. For detachment, 250 µl Accutase solution (Capricorn) were added to the cells and incubated for 5 min at 37 °C. The cells were then collected in PBS by stringent up and down pipetting followed by a washing step in PBS at 500 × g for 5 min at RT. Up to 1.0 × 10^5^ cells were incubated in 20 µl PBS containing 1% FCS for 20 min at RT to block non-specific binding sites. Cells were stained with fluorescently-labeled antibodies directed against CD14 (#301803, 400 ng/sample), CD45 (#304011, 400 ng/sample), CD16 (#302005, 400 ng/sample), CD11b (#301305, 400 ng/sample), CD163 (#333605, 400 ng/sample), HLA-DRB1 (#362303, 400 ng/sample), CD206 (#321106, 400 ng/sample) CD86 (#305417, 400 ng/sample), CD80 (#305219, 200 ng/sample), CD51 (#327907, 400 ng/sample, all from Biolegend), or the respective isotype controls at the same concentration (#400207, #400121, #400110, #400331, #400113 from Biolegend) for 20 min at RT. Fluorescence was recorded at the FACSymphony A1 flow cytometer (BD) and data was analyzed with FlowJo (v10.7, BD).

### Immunofluorescence

Osteoclasts, which were seeded on glass coverslips, were washed twice with PBS and fixed in 4% Paraformaldehyde (PFA) for 15 min at RT. Cells were incubated in PBS containing 0.3% BSA and 0.05% Saponin for 30 min to block non-specific binding sites and permeabilize the cells. To visualize the actin cytoskeleton, cells were stained with FITC-conjugated Phalloidin (1:1000, #P5282, Sigma) for 2 h at RT. Samples were mounted in mounting medium with DAPI (#ab104139, Abcam) and osteoclasts visualized on a BZ-X800 fluorescence microscope (Keyence) at 10 × magnification. High resolution images were obtained using confocal microscopy at the LSM 800 Airy Scan confocal microscope (Zeiss). Quantification of the multinuclear cells was performed with ImageJ (v1.5.3).

### TRAP staining

The staining for tartrate-resistant acid phosphatase (TRAP) in osteoclasts was performed with the Leukocyte Acid Phosphatase kit (Sigma-Aldrich) according to the manufacturer's protocol. Signals were visualized on an IX83 microscope (Olympus) with a 10 × objective.

### Bone resorption

To measure the capacity of osteoclasts to dissolve bone mineral, cells were either directly seeded and differentiated in 24-well plates coated with a proprietary synthetic inorganic bone mimetic matrix (Corning) or differentiated to macrophages for 7 days prior to seeding and terminal differentiation on the matrix. The wells had been equilibrated with growth medium prior to cell seeding. After a total differentiation period of 14 days, the wells were washed once with PBS and treated with 5% sodium hypochlorite for 5 min at RT. After a second washing step in PBS, the wells were dried and imaged via brightfield imaging (AE31E, Motic). To measure the capacity of osteoclasts to degrade organic bone matrix, cells were seeded and differentiated as described above on bovine cortical bone slices (FisherScientific) which had been washed twice in PBS and equilibrated in culture medium for at least 1 h prior to cell seeding. At day 14, medium was changed and cells incubated for another 72 h. The supernatant was collected and used for the determination of type I collagen degradation products using the CrossLaps for Culture ELISA (ids) according to the manufacturer’s instructions. The bone slices were either used for electron microscopy or toluidine blue staining to visualize the resorption pits. To this end, cells were disrupted by treatment with 1% Triton X-100 in H_2_O for 15 min and slices washed once in dH_2_O prior to staining for 4 min in 1% toluidine blue + 1% sodium borate 10-hydrate in H_2_O. Slices were washed extensively in H_2_O and imaged at the BZ-X800 microscope (Keyence). The pit area was quantified using ImageJ (v1.5.3).

### Electron microscopy

Bone slices were fixed in 2% (v/v) formaldehyde and 2.5% (v/v) glutaraldehyde in 100 mM cacodylate buffer, pH 7.4, at 4 °C overnight. After washing in PBS, samples were post-fixed in 0.5% (v/v) osmium tetroxide and 1% (w/v) potassium hexacyanoferrate (III) in 0.1 M cacodylate buffer for 2 h at 4 °C followed by washing with dH_2_O. After dehydration in an ascending ethanol series from 30 to 100% ethanol, specimens were two times incubated in propylene oxide each for 15 min and embedded in Epon using flat embedding molds. Ultrathin sections were cut with an ultramicrotome, collected on copper grids and negatively stained with 2% uranyl acetate for 10 min. Electron micrographs were taken at 60 kV with a Veleta camera system in combination with the Radius software system (emsis, Muenster, Germany).

### Cell viability assay

Cell viability of osteoclasts was analyzed with the Annexin V-FITC Apoptosis Detection Kit with 7-AAD (#640922, Biolegend) according to the manufacturer’s instructions. Fluorescence was recorded on a FACSymphony A1 flow cytometer (BD) and data was analyzed with FlowJo (v10.7, BD).

### Western blotting

Osteoclasts and their precursors were lysed in RIPA buffer (50 mM Tris, 150 mM NaCl, 0.1% SDS, 0.5% sodium deoxycholate, 1% Triton X-100, pH 7.2) supplemented with both protease (Sigma) and phosphatase (Roche) inhibitors. To determine the protein concentration, a Lowry assay (Bio-Rad) was performed. 20 µg of cell lysate were separated by SDS-PAGE (10% gel) and transferred onto a nitrocellulose membrane. To block non-specific binding sites, membranes were incubated in TBST (137 mM NaCl, 20 mM Tris pH 7.6, 0.1% (v/v) Tween-20) + 3% bovine serum albumin (BSA) for 1 h at RT. Subsequently, membranes were incubated with primary antibodies against CD51 (#sc-376156, 1:500), CD61 (#sc-365679, 1:500), MMP9 (#sc-393859, 1:500), RANK (#sc-374360, 1:1000), Cathepsin K (#sc-48353, 1:1000, all from Santa Cruz) and Tubulin (#11224–1-AP, 1:1000, Proteintech) overnight at 4 °C followed by incubation with the corresponding HRP-coupled secondary antibody (#7074 or #7076, 1:10,000, cell signaling) for 1 h at RT. The chemiluminescent signals were detected with ECL West Pico on a ChemoStar imager (Intas).

### RNA isolation and quantitative real-time PCR (qPCR)

Total RNA was isolated from the cells using the High Pure RNA isolation kit (Roche) according to the manufacturer’s instructions. 1 µg of RNA was reversely transcribed to cDNA with the iScript cDNA synthesis kit (Bio-Rad). Gene expression was measured by standard SYBR green detection on a QIAquant® 384 (Qiagen) using 10 ng of cDNA per sample. Primer sequences (5'-3') are as follows: *CTSK (*forward: TGTACCCTGAGGAGATACTG, reverse: CCAAATTAAACGCCGAGAG), *TNFRSF11A (*forward: GTACCAGTGAGAAGCATTATGAG, reverse: GAGGTAGTAGTGCATTTAGAAGAC), *MMP9 (*forward: ACCTGAGAACCAATCTCAC, reverse: GTAACCATAGCGGTACAGG), *ITGAV* (fw: CATTCTACTTGACTGTGGTG, rv: TTCTCCTTGATTCTGAGCC), *ACP5 (*forward: TGCAAGACATCAATGACAAGAG, reverse: CGGTCAGAGAATACGTCCT), *DCSTAMP* (fw: CAAAGATTCATTTCTGGCTTCC, rv: AATAGTGACTGCCATCCTAGAC), *NFATC1* (fw: TCCTCTCCAACACCAAAGTC, rv: AAGTTCAATGTCGGAGTTTCTG), *CA2* (fw: GATCAAGCAACTTCCCTGAG, rv: ACTGAATCAATCTGTAAGTGCC), *ATP6V0A1* (fw: CGGGACATGATTGACTTAGAG, rv: CCTGGTTTGTGTTGATTTCCT), *MMP14* (fw: GAAGGATGGCAAATTCGTC, rv: GAGCAGCATCAATCTTGTC), *MMP9* (fw: ACCTGAGAACCAATCTCAC, rv: GTAACCATAGCGGTACAGG), *GNB2L1* (forward: AACCCTATCATCGTCTCCT, reverse: CAATGTGGTTGGTCTTCAG), *HPRT1* (forward: TATGCTGAGGATTTGGAAAGG, reverse: CATCTCCTTCATCACATCTCG). Gene expression was normalized on the two housekeeping genes *HPRT1* and *GNB2L1*.

### RNA-Sequencing (RNA-Seq) analysis

Sequencing was performed at the Core Facility Genomics of the Medical Faculty Muenster. Quality and integrity of total RNA was controlled on Agilent Technologies 2100 Bioanalyzer (Agilent Technologies). Poly(A) mRNA were purified from 300 ng total RNA using Poly(A) mRNA Magnetic Isolation module Kit (NEB E7490L, New England Biolabs). The RNA-Seq library was prepared with NEBNext Ultra™ II Directional RNA Library Prep Kit for Illumina (New England Biolabs). The libraries were sequenced on Illumina NextSeq 2000 using NextSeq2000 P3 Reagent Kit with single-end reads with a length of 72 base pairs (bp) and an average of 27.9 M reads per RNA sample. Pre-processing of the raw reads involved adapter trimming using TrimGalore (v0.6.6) in conjunction with Cutadapt (v4.4). The specific adapter sequence targeted for removal during trimming was AGATCGGAAGAGCACACGTCTGAACTCCAGTCAC. Subsequently, reads shorter than 30 bp were filtered out. The trimmed reads were mapped to the CHM13 reference human genome (v2.0) and the alignment was performed using STAR (v2.7.3a). Gene-level counts were generated using featureCounts from subread (v2.0.2), incorporating strand-specific information (reverse strandedness). The resulting raw reads count table indicated an average alignment rate of 74.6%. Differential expression analysis was carried out using the ALDEx2 pipeline [16]. This analytical approach ensures robust exploration of differential expression patterns and provides a comprehensive understanding of the compositional nature of the RNA-Seq data in the context of this study.

### Statistical analysis

All experiments were performed with at least three biological replicates. Statistical significance was calculated with GraphPad Prism (v9.2.0) using an unpaired t-test for comparison of two groups or a one-way ANOVA with Fisher’s LSD test for comparison of multiple groups, unless stated otherwise. *P*-values < 0.05 were considered as significant (****p < 0.0001, ***p < 0.001, **p < 0.01, *p < 0.05, ns = not significant). All figures in the current study were generated with GraphPad Prism (v9.2.0) or OMERO (v5.14.1) in case of the immunofluorescence images.

## Resource list

- PBS (Pan-Biotech, Germany, P04-36500)
- EDTA (EDTA, PanReac AppliChem, Germany)
- Pancoll (Pan Biotech, Germany, P04-60500)
- RPMI-1640 w/o Phenolred (Gibco, Germany, 11835030)
- RPMI-1640 (PanBiotech, Germany, P04-16500)
- FCS (7BioScience, Germany, 7-FBS-11A)
- Percoll (FisherSientific, Germany, 11530734)
- 10 × PBS (Gibco, Germany, 11520486)
- FEP Teflon bags (CellGenix, Germany, 003300)
- Polystyrol cell culture plates 6-well (Sarstedt, Germany, 83.3920)
- Polystyrol cell culture plates 24-well (Sarstedt, Germany, 83.3922)
- M-CSF (Immunotools, Germany, 1020879)
- RANKL (Peprotech, Germany, 310–01)
- Accutase (Caprichorn, Germany, ACC-1B)
- CD14-FITC (Biolegend, Germany, 301803)
- IgG2a-FITC (Biolegend, Germany, 400207)
- CD45-APC (Biolegend, Germany, 304011)
- IgG1-APC (Biolegend, Germany, 400121)
- CD51-FITC (Biolegend, Germany, 327907)
- CD16-FITC (Biolegend, Germany, 302005)
- IgG1-FITC (Biolegend, Germany, 400110)
- CD11b-PE (Biolegend, Germany, 301305)
- CD163-PE (Biolegend, Germany, 333605)
- CD206-PE (Biolegend, Germany, 321106)
- HLA-DRB1-PE (Biolegend, Germany, 362303)
- CD80-APC (Biolegend, Germany, 305219)
- CD86-Pacific Blue (Biolegend, Germany, 305417)
- IgG2b-Pacific Blue (Biolegend, Germany, 400331)
- IgG1-PE (Biolegend, Germany, 400113)
- Flow cytometer (BD Biosciences, Germany, FACSymphony A1)
- PFA (Santa Cruz, Germany, sc-281692)
- BSA (Roth, Germany, 80763)
- Saponin (PanReac AppliChem, A2542.0100)
- FITC-Phalloidin (Sigma Aldrich, Germany, P5282)
- DAPI (Abcam, Germany, ab104139)
- Fluorescence microscope (Keyence, Germany, BZ-X800)
- Fluorescence microscope (Olympus, Germany, IX83)
- Acid Phosphatase, Leukocyte (TRAP) Kit (Sigma Aldrich, Germany, 387A)
- Bone resorption assay plate 24 (Corning, Germany, CLS3590)
- Bovine cortical bone slices (FisherScientific, NC1309388)
- CrossLaps for Culture ELISA (ids, Germany, AC-07F1)
- Triton X-100 (Th. Geyer, Germany, 80.590.500)
- Toluidine blue (Sigma, Germany, T3260)
- Sodium borate 10-hydrate (Sigma, Germany, S9640)
- Sodium hypochlorite (Sigma Aldrich, Germany, 1056142500)
- Inverted light microscope (Motic, Germany, AE31E)
- Annexin V Apoptosis Detection Kit with 7AAD (Biolegend, Germany, 640922)
- Protease Inhibitor Cocktail (Sigma Aldrich, Germany, P-8340-1ML)
- PhosStop Phosphatase Inhibitor (Roche, Germany, 1024238)
- Dc Lowry protein assay kit II (Bio-Rad, Germany, 5000112)
- RANK antibody (Santa Cruz Biotechnology, Germany, sc-374360)
- Cathepsin K antibody (Santa Cruz Biotechnology, Germany, sc-48353)
- ITGɑV antibody (Santa Cruz Biotechnology, Germany, sc-376156)
- ITGβ3 antibody (Santa Cruz Biotechnology, Germany, sc-365679)
- MMP9 antibody (Santa Cruz Biotechnology, Germany, sc-393859)
- Tubulin antibody (Proteintech, Germany, 11224–1-AP)
- HRP-coupled secondary anti-mouse antibody (Cell Signaling, Germany, 7076S)
- HRP-coupled secondary anti-rabbit antibody (Cell Signaling, Germany, 7074S)
- High pure RNA isolation kit (Roche, Germany, 11828665001)
- cDNA synthesis kit (Bio-Rad, Germany, 1080854)
- qPCR Cycler (Qiagen, Germany, QIAquant® 384)

## Results

### Experimental setup for osteoclast differentiation

Typically, monocytes isolated from donor blood are seeded into cell culture plates and differentiated to osteoclasts through the addition of M-CSF and RANKL (Fig. 1A). The cells remain in the plates throughout the whole differentiation period. As our previous study has revealed that monocyte-to-macrophage differentiation can be significantly improved in terms of yield upon cultivation of the cells in Teflon cell culture bags [15], we tested whether the generation of osteoclasts from primary monocytes can be equally improved with this approach. To this end, monocytes were maintained in the presence of M-CSF in Teflon bags throughout the first half of the differentiation period (7 days), where no medium change is required. After 7 days, the cells were harvested from the bags and seeded in cell culture plates in the presence of M-CSF and RANKL to induce terminal osteoclast differentiation (Fig. 1B).

To characterize the precursor cells used for osteoclast generation in this study, we performed a flow cytometric characterization of the primary human monocyte preparations that had been isolated from healthy donor blood by double density gradient centrifugation. The analysis confirmed that around 80% of the cells belonged to the monocyte lineage (Fig. 1C + D), whereas lymphocytes and granulocytes made up only a minor part of the samples. The monocytes were further characterized for the expression of common surface markers, including CD14, CD16, CD11b, and HLA-DRB1 (Fig. 1E). Monocytes can be classified into different subsets based on their marker expression: classical monocytes are CD14^++^CD16^−^, intermediate monocytes are CD14^++^CD16^+^ and non-classical monocytes are CD14^+^CD16^+^ [17]. The cells in our study showed a comparatively high expression of CD14 (mean ± SD: 89.86 ± 1.23% positive cells), CD11b (mean ± SD: 90.72 ± 4.4% positive cells) and HLA-DRB1 (mean ± SD: 53.27 ± 3.79% positive cells), whereas CD16 (mean ± SD: 5.5 ± 2.0% positive cells) was found only on a minor population of monocytes.

### Characterization of macrophages differentiated in Teflon bags or cell culture plates

**Fig. 2:**
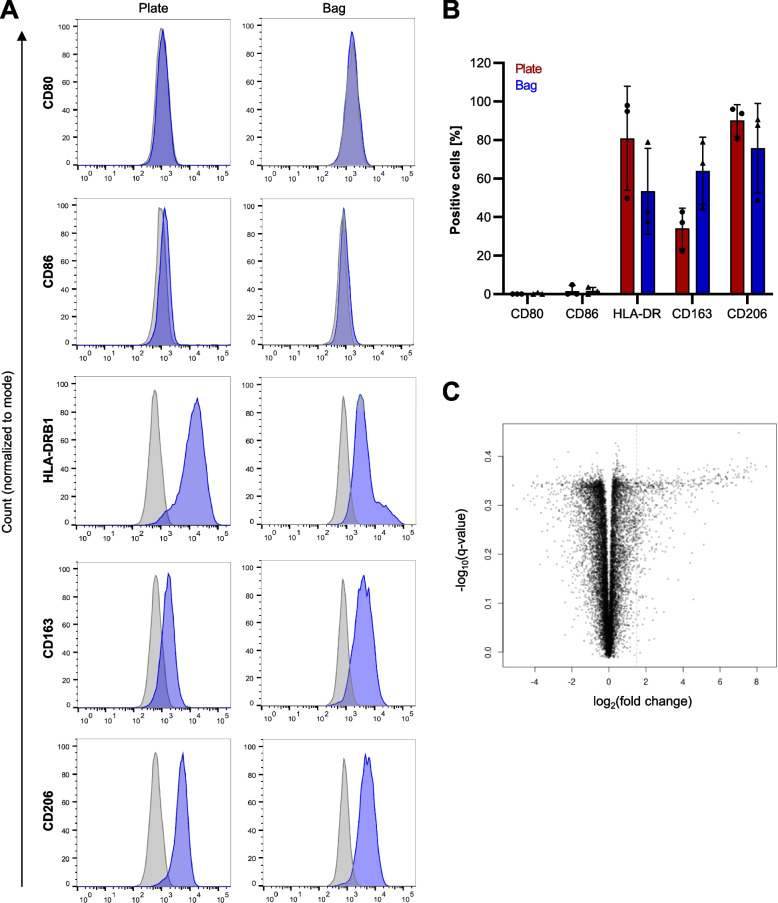
Macrophages from Teflon bags and cell culture plates do not differ significantly in their gene and marker expression profile. **A** + **B**, Flow cytometry: Expression of common macrophage surface marker proteins on cells differentiated either in regular polystyrene cell culture plates or Teflon bags from matched donors. Shown is one representative histogram per marker (**A**) and the corresponding quantification (**B**). Blue: antibody-of-interest, grey: isotype control. **C** RNA-Seq: Volcano plot comparing the gene expression profile in macrophages differentiated in Teflon bags and polystyrene cell culture plates. Black dots denote genes with no significant changes. None of the genes reached the significance threshold at *p* = 0.05 [–log10 (1.30)]

As a first step in osteoclastogenesis, monocytes were differentiated to macrophages either in Teflon bags (novel protocol) or in regular polystyrene cell culture plates (standard protocol) for 7 days. Depending on the environment and polarization status, macrophages can express distinct markers that are either associated with a pro-inflammatory (e.g. CD80, CD86, HLA-DRB1) or anti-inflammatory (e.g. CD163, CD206) phenotype. To investigate whether the differentiation process in Teflon bags influences the polarization and activation status of the cells, macrophages obtained by both protocols were compared by flow cytometry for the expression of these markers (Fig. 2A + B). In either case, macrophages were positive for CD163 (mean ± SD, *n* = 3: Plate 34.1 ± 10.5%, Bag 64.0 ± 17.5%), CD206 (mean ± SD, *n* = 3: Plate 90.1 ± 8.2%, Bag 75.7 ± 23.3%) and HLA-DRB1 (mean ± SD, n = 3: Plate 80.9 ± 27.0%, Bag 53.4 ± 22.3%). The surface marker CD80 was not detectable in neither population and CD86 was only expressed on some residual cells (mean ± SD, *n* = 3: Plate 1.6 ± 2.8%, Bag 1.7 ± 1.7%). Despite the comparatively high expression of HLA-DRB1, the characterization suggested that macrophages tended to differentiate towards an alternatively activated M2-like phenotype with both protocols. To gain a more comprehensive insight into the influence of the cultivation system on the cells, we performed an in-depth comparison of the macrophage transcriptome. To this end, monocytes from five healthy donors were differentiated to macrophages with either the standard or the novel protocol and matched samples were subjected to RNA-Seq. However, we did not detect any differentially expressed genes (Fig. 2C). Taken together, these observations indicated that the cultivation in Teflon bags has no major influence on macrophage gene or surface marker expression.

### Osteoclastogenesis can be improved by macrophage differentiation in Teflon bags, high cytokine concentrations and an intermediate cell density

**Fig. 3:**
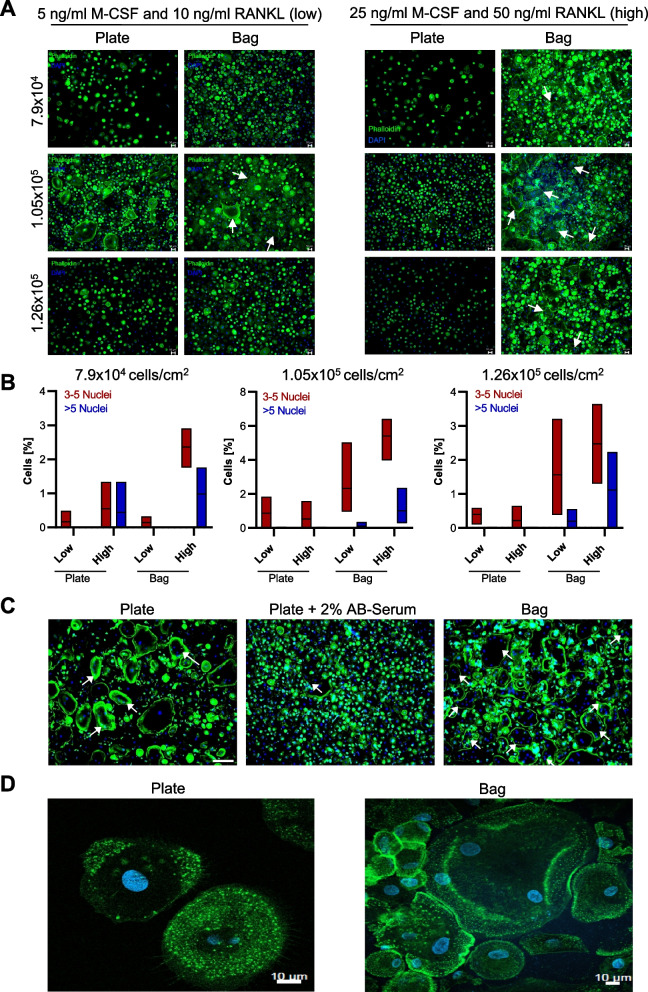
Cell density and concentration of M-CSF and RANKL are crucial for osteoclast differentiation. **A** + **B** Immunofluorescence: Isolated monocytes were seeded at different cell densities (indicated as cells/cm^2^) and stimulated with either a low (5 ng/ml M-CSF, 10 ng/ml RANKL) or high (25 ng/ml M-CSF, 50 ng/ml RANKL) concentration of cytokines. Mature osteoclasts were assessed by staining for F-Actin (green) and DAPI (blue) (**A**). White arrows point at cells with ≥ 3 nuclei (scale bar: 50 µm). The proportion of multinucleated cells was counted and compared between the different conditions (Boxplots depict the mean (line) and the 25–75 percentiles (box) of 3 data points) (**B**). **C** Immunofluorescence: Isolated monocytes were differentiated to osteoclasts according to the standard (plate) or novel (bag) protocol. Macrophage differentiation in plates was performed with cells either cultivated in their normal growth medium or in medium additionally supplemented with 2% AB serum. White arrows point at cells with ≥ 3 nuclei. **D** Confocal microscopy: Monocytes (2.0 × 10.^5^) from cell culture plates or Teflon bags were seeded on glass coverslips, differentiated to osteoclasts in the presence of high concentrations of M-CSF and RANKL and stained for F-Actin (green) and DAPI (blue) (scale bar: 10 µm)

As osteoclastogenesis is a critical, multistep process, which can be influenced by various experimental parameters, we aimed to optimize cell density as well as the concentration of the two critical cytokines for osteoclast differentiation, M-CSF and RANKL, in order to maximize the generation of mature osteoclasts. To this end, macrophages differentiated either in plates or in bags were seeded at three different densities (7.9 × 10^4^, 1.05 × 10^5^, 1.26 × 10^5^ cells/ cm^2^) in 24-well plates and treated with either 5 ng/ml M-CSF and 10 ng/ml RANKL (low concentration) or 25 ng/ml M-CSF and 50 ng/ml RANKL (high concentration) for inducing osteoclast formation. As mature osteoclasts can be identified by their multinuclearity, immunofluorescence analysis of the cell shape and the number of nuclei was conducted as a readout for efficient osteoclast differentiation (Fig. 3A). Only cells with three or more nuclei were considered as mature osteoclasts. Immunofluorescence analysis and the corresponding quantification revealed that initial macrophage differentiation in bags as well as the addition of high concentrations of M-CSF and RANKL yielded the highest proportion of mature osteoclasts (Fig. 3B). In comparison, we observed that only minimal numbers of multinucleated, mature osteoclasts were obtained with the standard protocol from macrophages that had been differentiated in polystyrene plates. Among the three tested macrophage concentrations, an intermediate cell density of 1.05 × 10^5^ cells per cm^2^ resulted in the highest number of mature osteoclasts. Hence, this concentration was used for all further analyses. As the culture medium for monocytes in the Teflon bags additionally contains 2% human AB serum to support cell growth, we tested whether the addition of this factor to monocytes cultured in cell culture plates would support their differentiation to macrophages, and terminal osteoclasts. However, immunofluorescence analysis of the cells indicated that AB serum had no beneficial effect on the differentiation process (Fig. 3C), suggesting that the improved osteoclastogenesis results from the cultivation in the Teflon bags and not from differences in the culture medium. High-resolution confocal microscopy images confirmed the presence of multinuclear cells as well as the presence of the characteristic F-actin ring in osteoclasts derived from the novel approach (Fig. 3D). In comparison, the vast majority of cells differentiated with the standard plate-based protocol did not harbor these features and rather tended to show actin-reactive dot-like podosomes indicative of an early stage of osteoclast differentiation (Fig. 3D). Taken together, these results suggested that based on cell morphology, the generation of osteoclasts from macrophages differentiated in Teflon bags at an intermediate cell density and in the presence of high concentrations of M-CSF and RANKL yielded the highest number of mature osteoclasts.

### Osteoclast derived from Teflon bags remain viable and show higher bone resorption

**Fig. 4:**
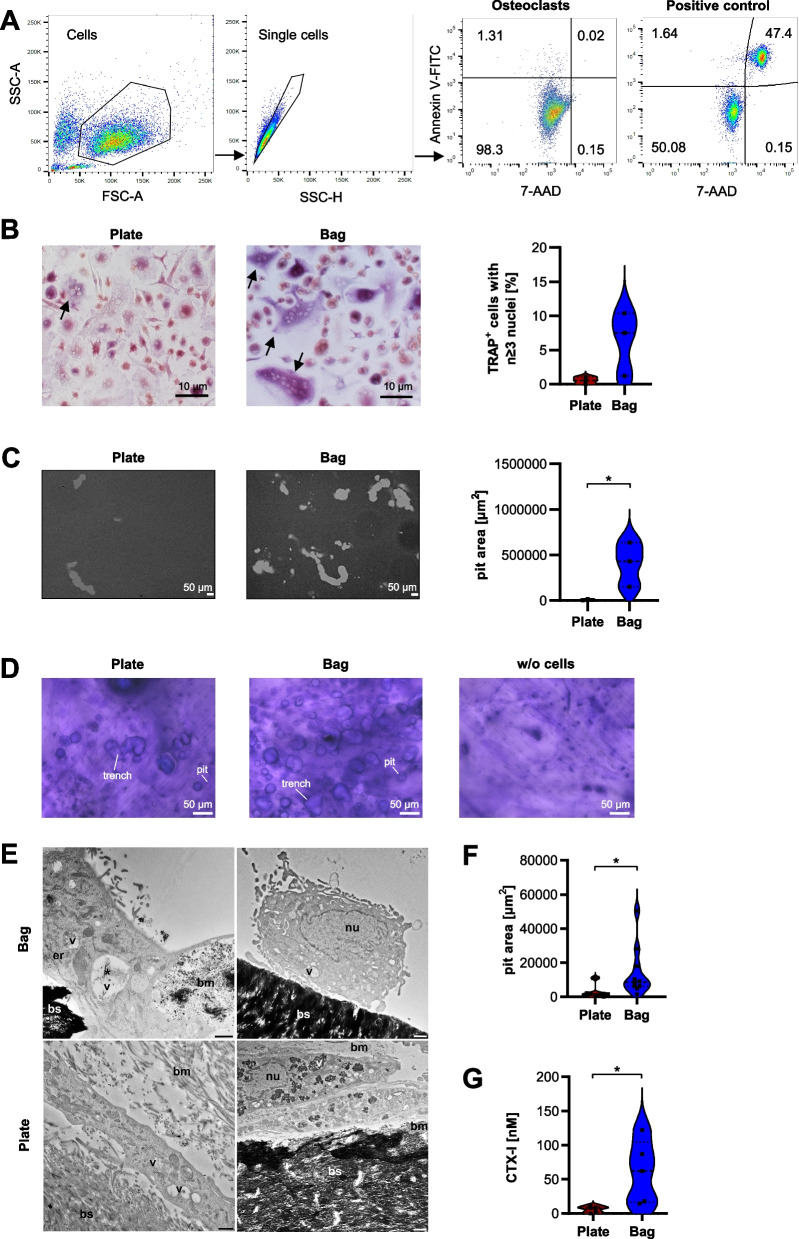
Osteoclasts differentiated from Teflon-coated bag-derived macrophages remain viable and show increased TRAP-positivity and bone resorption. **A** Flow cytometry: Gating strategy used to identify osteoclasts differentiated from Teflon bag-derived macrophages. Cell viability was assessed by staining for 7-AAD and Annexin V-FITC. A 1:1 mixture of untreated and heat-incubated (10 min at 95 °C) osteoclasts was used as positive control. Shown is one representative scatter plot out of n > 3 donors. **B** Monocytes (2.0 × 10^5^) differentiated to osteoclasts in the presence of high concentrations of M-CSF and RANKL in cell culture plates or in Teflon bags were stained for TRAP activity (scale bar: 10 µm) and visualized by microscopy. Black arrows indicate TRAP-positive and cells with ≥ 3 nuclei. The corresponding quantification of TRAP-positive cells is shown on the right. **C** Bone mineral resorption of osteoclasts on synthetic inorganic bone matrix is shown in representative bright-field images and corresponding quantification on the right. **D** Bone resorptive activity of osteoclasts on bovine cortical bone slices was visualized by toluidine blue staining. **E** Representative electron microscopy images of osteoclasts from two distinct donors seeded on bone slices. nu = nucleus, er = endoplasmic reticulum, bs = bone slice, bm = bone matrix (partly degraded), v = vacuole, asterisk = not degraded collagen fibrils; scale bar: 1 µm. **F** Quantification of the pit area formed by osteoclasts differentiated on bone slices. **G** The release of type I degradation products into the supernatant of osteoclasts cultured on bone slices was measured by ELISA

In order to confirm that the osteoclasts generated with the novel protocol are indeed viable and active, we analyzed the viability of non-permeabilized cells by co-staining of Annexin V, which binds externalized phosphatidylserine (PS), a marker for the early phases of apoptosis, and 7-AAD, a DNA-intercalating dye that is only able to enter late-stage apoptotic cells that have lost their membrane integrity. Cells which were positive for both markers were considered as apoptotic. A mixture of osteoclasts heated at 95 °C for 10 min (50%) and non-heated osteoclasts (50%) was used as positive control. Flow cytometric analysis of naïve osteoclasts revealed that 94.47 ± 7.25% (mean ± SD, *n* = 3) of the cells harvested from the novel protocol were negative for both markers, and thus viable (Fig. 4A). This suggests that the novel method for osteoclast differentiation does not affect cell viability. To confirm that the generated osteoclasts are also functionally active, we measured the activity of TRAP and compared it to osteoclasts obtained from the standard protocol (Fig. 4B). Indeed, we observed more TRAP-positive osteoclasts when cells were differentiated from Teflon bag-derived macrophages. In order to analyze whether this difference was also reflected in osteoclast functionality, we measured their bone resorption ability. To this end, cells were seeded into cell culture plates coated with a synthetic inorganic bone matrix and the resorbed area was imaged and quantified (Fig. 4C). Intriguingly, osteoclasts differentiated with the novel protocol showed a more than 40-fold increase in their resorption area in comparison to osteoclasts derived from plates. To confirm that the cells are not only able to degrade mineral, but also organic bone matrix, they were seeded on bovine cortical bone slices and bone resorption was evaluated at day 17. We observed that osteoclasts differentiated from plate- or bag-derived macrophages both had formed pits and trenches (Fig. 4D), the latter being indicative of greater depth and higher collagenolysis [18]. Electron microscopy revealed that the cells interacted with the surface of the bone slice and showed detectable signs of bone degradation, including partly degraded bone matrix as well as collagen fibrils (Fig. 4E). Quantification of the area of the formed pits as well as type I collagen degradation products in the supernatant demonstrated significantly elevated resorptive activity for the osteoclasts differentiated from bag-derived macrophages (Fig. 4F + G). These observations fit to the increased number of TRAP-positive cells suggesting that osteoclasts obtained from the novel protocol differentiate more efficiently in terms of yield, maturity and resulting bone resorption in vitro. Since a normalization on osteoclast cell counts was not possible due to technical reasons, it remains to be understood whether the increase in bone resorption arises from a higher number of terminally differentiated osteoclasts from bag-derived macrophages or from a higher resorptive activity of the cells per se.

### Osteoclasts derived from Teflon bags express the typical markers

**Fig. 5:**
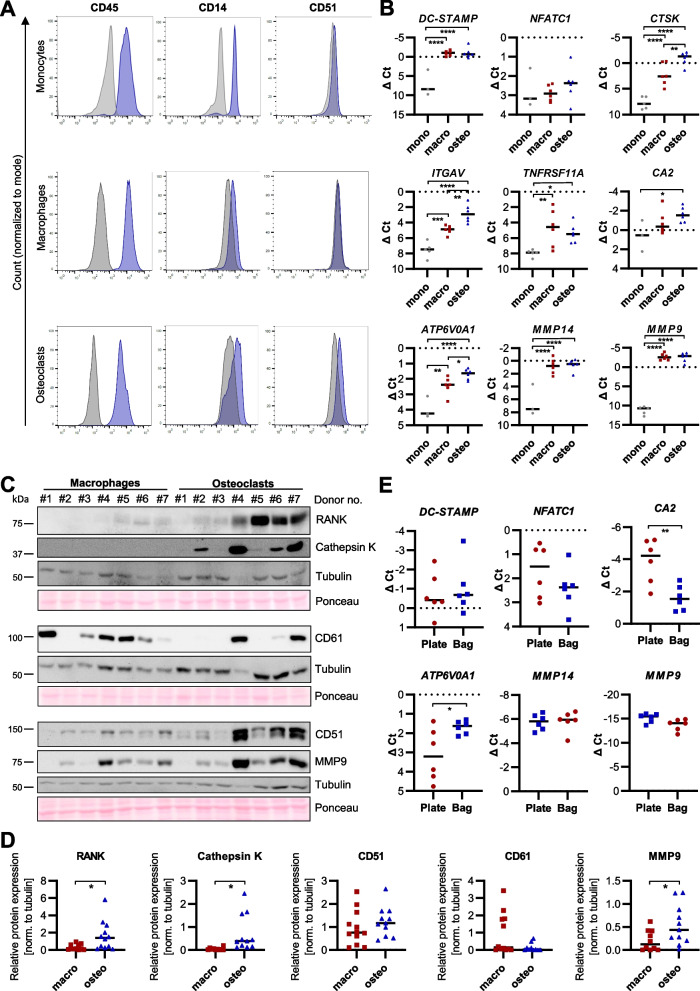
Osteoclasts differentiated from Teflon bag-derived macrophages express typical osteoclast markers. **A** Flow cytometry: Expression of surface marker proteins (CD45, CD14, CD51) was assessed on monocytes, macrophage and bag-derived osteoclasts on n > 3 donors. Shown is one representative histogram per staining. Blue: antibody-of-interest, grey: isotype control. **B** Expression of known osteoclast marker genes was measured by qPCR (line at median). **C** + **D** Expression of selected osteoclast marker proteins was analyzed via western blotting in whole cell lysates (20 µg) of macrophages and osteoclasts (**C**). Signals were quantified by densitometry and normalized to Tubulin expression (line at median) (**D**). Outliers were identified and excluded according to the ROUT method (Q = 1%). **E** Expression of early- (*DCSTAMP, NFATC1, CA2*) and late-stage (*ATP6V0A1, MMP9, MMP14*) osteoclast markers was measured by qPCR in osteoclasts differentiated in cell culture plates or from Teflon bag-derived macrophages (line at median)

The osteoclasts differentiated with the novel approach seemed to fulfill the morphological and functional characteristics for mature osteoclasts. In order to further characterize the differentiation state of the cells, we used flow cytometry to measure the expression of typical osteoclast surface markers on monocyte and macrophage precursors, and osteoclasts derived from macrophages, which were differentiated in Teflon bags. The marker panel included CD45 and CD14 as markers for the hematopoietic or myelomonocytic lineage, respectively, as well as the osteoclast-specific surface protein CD51 (Integrin alpha V/*ITGAV*). As expected, all three cell populations were positive for CD45, and CD14 (Fig. 5A). Up to 5% of osteoclasts carried CD51, while the marker was not found on macrophages and on only 2% of monocytes (Fig. 5A). Using qPCR, we furthermore measured the expression of typically known osteoclast marker genes, including genes expressed early during osteoclastogenesis, such as *DC-STAMP*, *NFATC1, CA2* (carbonic anhydrase 2), or *CTSK *(Cathepsin K)*,* and genes expressed in later developmental stages, including the vitronectin receptor (CD51/*ITGAV*), receptor activator of NF-κB (RANK/*TNFRSF11A*), the V-type proton ATPase subunit a 1 (*ATP6V0A1*) and the matrix metalloproteinases (MMPs) *MMP9* and *MMP14*. The qPCR analysis revealed that all osteoclast marker genes showed higher expression levels in macrophages and osteoclasts compared to their monocyte precursors, indicating cellular differentiation to the macrophage-osteoclast lineage (Fig. 5B). Osteoclasts were significantly enriched in *CTSK*, *ITGɑV*, and *ATP6V0A1*, which have been preferentially observed in multinucleated cells [19], compared to their macrophage counterparts.

To validate the expression of the osteoclast markers on the protein level, we measured RANK, Cathepsin K, MMP9, CD51, as well as its interaction partner CD61 (Integrin β3) in osteoclasts and macrophages from matched donors by immunoblotting (Fig. 5C + D). In line with the qPCR results, RANK was equally expressed in both cell populations. Cathepsin K and CD51 were enriched in osteoclasts, but were also detectable in macrophages, albeit to a lesser extent and in fewer samples. CD51 forms a heterodimer with CD61 which is required to constitute the vitronectin and osteopontin receptor which is important for osteoclast function [20]. CD61 was expressed in three osteoclast preparations, but only one corresponding macrophage sample. MMP9 was found more prominently in osteoclasts compared to macrophages hinting at a significantly higher expression of the functional vitronectin receptor in the bone-resorbing cells. Of note, our analyses revealed a high degree of inter-donor variability regarding marker expression, indicating a high heterogeneity among primary human osteoclasts and macrophages (Fig. 5B-D). Taken together, the characterization results confirmed that the novel protocol indeed yielded mature osteoclasts that expressed typical markers related to the osteoclast phenotype and function.

Among these markers, in particular *DC-STAMP* and *NFATC1* are considered as master regulators for inducing osteoclastogenesis [21, 22], while *ATP6V0A1, CA2, MMP9 and MMP14* are essential for the bone resorptive activity of the cells [23, 24]. Since our results had identified osteoclasts differentiated from Teflon bag-derived macrophages to morphologically resemble mature osteoclasts and to harbor a higher functional activity compared to plate-derived osteoclasts, we measured the expression of the selected genes in both cell populations (Fig. 5E). While plate-derived osteoclasts showed a trend for a still high expression of the early differentiation marker *NFATC1*, we did not observe significant changes in *DC-STAMP.* Likewise, both *MMP9* and *MMP14* were highly expressed in the two cell populations. Interestingly, *CA2* was abundant in both osteoclast populations, albeit at higher levels in the conventionally differentiated osteoclasts. In contrast, *ATP6V0A1* was detectable at higher levels in osteoclasts obtained with the novel approach from Teflon bags and only minimally expressed in plate-derived osteoclasts. Since *NFATC1* and *CA2* are already induced during the early stages of osteoclastogenesis [22, 25], in summary our results could suggest that plate-derived human osteoclasts rather resemble an early cell differentiation stage while osteoclast generation via the Teflon bags seems to yield more mature cells.

## Discussion

Currently, there is an unmet need for protocols that allow the efficient, large-scale and reproducible isolation of human osteoclasts for further in vitro studies. Here, we have established a novel approach that addresses this problem. The protocol presented in this study is based on the initial culture of the cells in Teflon bags, which not only allows their flexible reseeding after 7 days of culture, but also significantly improves the bone resorption activity of the obtained osteoclasts.

The multistep process of osteoclastogenesis is highly complex and difficult to mimic in vitro. Therefore, we have put our focus on testing the optimal cell density and cytokine concentration, two factors that are essential for an efficient osteoclast differentiation. In line with recent results from Remmers et al*.* [13], we observed that the cell density has a major effect on the efficiency of osteoclastogenesis and measured the most efficient differentiation at an intermediate cell number. The fact that seeding density significantly impacts osteoclast differentiation has not only been observed for osteoclasts of human origin, but also for differentiation protocols based on murine/rat origin [26, 27], thus indicating that cell density is a critical parameter in determining efficient osteoclastogenesis across various culture models. Another critical factor is the concentration of the two cytokines that reprogram monocytes towards osteoclasts, M-CSF and RANKL. Confirming previously published data, our results demonstrate that high concentrations of these two factors are clearly superior in inducing efficient osteoclast differentiation [26, 28–30]. Some studies have applied an even higher concentration of RANKL (100 ng/ml) and observed a further increase in the number of TRAP-positive cells. In addition to M-CSF and RANKL, some studies used other supplements including dexamethasone or transforming growth factor-β1 (TGF-ꞵ1), however, these treatments were associated with the requirement for a longer differentiation period [30]. In our approach, no additional supplements were required, as M-CSF and RANKL treatment was sufficient to establish mature osteoclasts. Another advantage of our protocol is that 14 days of differentiation were sufficient for high osteoclasts yields.

The original cell population used for the differentiation workflow were PBMCs, a mixture of lymphocytes, monocytes and dendritic cells. Among these cells, the main osteoclast precursors are CD14^+^ monocytes, which have the highest osteoclastogenic potential [31]. Using a double density gradient-based protocol, we were able to obtain a preparation that contained mostly monocytes that showed a high expression of CD14. Although an enrichment for CD14^+^ monocytes by magnetic/fluorescence-activated cell sorting (MACS) might be reasonable to further enhance osteoclast yields, a priori positive selection for CD14^+^ precursors might affect cell differentiation due to the induction of different cellular phenotypes and the presence of residual beads in the culture system [32]. Indeed, Hornschuh and colleagues reported a significant decrease in monocyte functionality, cytokine release and viability after MACS-based CD14 sorting compared to negatively selected cells [33]. However, another study observed that negatively selected monocytes might be contaminated with high numbers of platelets and skewed towards a M2 polarization [34]. Due to the potential side effects of the selection procedure, we refrained from using such an approach in this study. In general, a careful evaluation of the influence of the chosen purification strategy on the phenotype of the generated osteoclasts should be performed prior to the use of these models for functional studies.

Next to the monocyte isolation procedure, gender- and age-related differences can influence osteoclast resorptive function as well as the expression of osteoclastogenesis-related genes [35, 36]. A limitation of this study is that neither the gender nor the age of the healthy blood donors used for PBMC isolation and osteoclast differentiation are known. However, as side-by-side comparisons of osteoclasts from plate- or bag-derived macrophages were always performed on matched samples from the same donor, the differences observed for differentiation efficiency, marker expression and function of the two differentiation approaches cannot arise from these two factors. It should furthermore be noted that using primary human cells, such as human PBMCs, we observed a high inter-donor heterogeneity, which could affect the osteoclastogenic potential and downstream analyses. Alternative osteoclast models, such as immortalized cell lines (e.g. THP1, RAW264.7) or primary cells from inbred mouse lines, promise more homogeneity, albeit at the risk of reduced translatability of the results and high costs for animal breeding.

The novel protocol presented in this study relies on the initial differentiation of monocytes to macrophages in Teflon bags prior to terminal osteoclast differentiation. These closed culture systems have initially been developed for the GMP-certified large-scale production of cells to minimize the risk of contamination. The FEP Teflon allows a comparable gas exchange, but possesses a lower stiffness, which more closely resembles the conditions found in human tissues, compared to polymers commonly used to manufacture cell culture vessels [37]. In contrast to the standard surface-treated hydrophilic polystyrene cell culture plates or flasks, the inherent Teflon surface of the bags is hydrophobic and thus reduces cell adhesion and spreading [38]. Consequently, the cells remain in suspension and can be easily harvested for downstream analyses. The first study to culture monocytes in suspension in a Teflon cell culture bag dates back to 1982 and found no major difference in morphology, anti-bacterial activity, or locomotion compared to uncultured cells [39]. In the meantime, several studies have reported the successful use of this cell culture system for the large-scale cultivation of human mesenchymal stem cells [40], early endothelial progenitor cells [41], or dendritic cells [42–44]. While data on Teflon bag-derived macrophages are scarce, several studies have observed that monocyte differentiation to dendritic cells in closed system cell culture bags is equivalent or superior in terms of functionality and yield to comparable cells cultured in regular cell culture plates [42–44]. In line, we did not observe major differences in cellular marker expression when comparing plate- and bag-derived macrophages suggesting that the difference in osteoclast differentiation efficiency and function might arise from the lower adhesion or the prolonged incubation in the culture medium in the Teflon bags. Our calculations have revealed that monocyte differentiation in regular cell culture plates requires 6.6-fold more culture medium, including the expensive growth factors, compared to the closed culture system which does not require any medium change for 7 days. Although the exact mechanism that results in the superior osteoclastogenesis of the novel protocol requires further investigation, the approach seems to be particularly feasible if a cost-effective large-scale production of human osteoclasts is required.

The two early osteoclastogenic genes *NFATC1* and *DC-STAMP* are considered as master regulators of osteoclastogenesis [21, 22]. The expression of the transcription factor *NFATC1* is induced via RANKL signaling, and in turn induces the expression of *DC-STAMP* [45]. *DC-STAMP* is required for cells to fuse between bilayers [46]. The still high levels of both markers in the generated osteoclasts might be due to the substantial proportion of mono- and binuclear cells, indicative of early differentiation phases that remain in the preparations even after 14 days of culture. Increased expression of *MMP9*, *MMP14* and *ATP6V0A1* has been associated with late stage osteoclastogenesis and all genes are essential for the bone resorption activity of osteoclasts [24, 47]. However, the expression of the MMPs is not osteoclast-specific but can also be detected in macrophage precursors, in which they influence macrophage function in immunity and inflammation via their ECM-remodeling capacities [48]. Therefore, it is not surprising that in this study both osteoclasts and macrophages expressed high levels of *MMP9* and *MMP14*. Instead, the late-stage osteoclast markers *ATP6V0A1* and *CTSK* were significantly enriched in osteoclasts compared to macrophages. Likewise, we observed an osteoclast-specific enrichment of CD51, the integrin-alpha subunit of the vitronectin receptor, while its interaction partner CD61 was equally expressed in macrophages and osteoclasts. The heterodimerization of CD51 and CD61 is a prerequisite for osteoclasts to bind to the bone surface and to exert their bone resorption activity [20]. Based on the expression of the selected markers, our results thus hint at the presence of mature osteoclasts differentiated with our novel approach.

The high levels of *CTSK*, *CA2*, *ATP6V0A1, MMP9 and MMP14* in osteoclasts derived from Teflon bags correlated with a high functional activity of the cells in bone resorption assays. Conversely, osteoclasts from the standard differentiation protocol showed significantly less bone resorption suggesting that they either largely remain in an early differentiation state or lack the expression of functionally relevant markers, which might hamper their bone resorption. Although the characterization of the cells indicated a high expression of *MMP9, MMP14* and *CA2* in the cells, the expression level of the V-ATPase subunit *ATP6V0A1* was comparatively low. Although *CA2* and *ATP6V0A1* act in concert to initiate bone resorption (Cappariello et al., 2014), the expression of the V-ATPase might be rate limiting for the resorptive activity of the osteoclasts. While these results might indicate that initial differentiation of macrophages in Teflon bags yields more mature osteoclasts compared to the standard plate-based approach, further studies on the expression of the distinct markers during osteoclast differentiation are needed to specify the exact developmental stage of the cells obtained with the different protocols.

## Conclusion

To the best of our knowledge, we show for the first time that macrophage differentiation in Teflon bags supports the generation of mature, and highly active human osteoclasts compared to the conventionally used method. The remarkably strong bone resorption activity of the osteoclasts derived from the novel method makes them highly suitable for studies with a clinical perspective, for instance screening for bone resorption inhibitors or co-culture models.

## Data Availability

The RNA-Seq data have been uploaded to the GEO repository under the identifier GSE248902. All data supporting the findings of this study are available within the article, or from the corresponding author upon reasonable request.

## Abbreviations

ATP6V0A1: V-type proton ATPase subunit a 1
CA2: Carbonic anhydrase 2
CTSK: Cathepsin K
DC-STAMP: Dendritic cell specific transmembrane protein
FCS: Fetal calf serum
FEP: Fluorinated ethylene propylene
HSCs: Hematopoietic stem cells
ITGAV: Integrin-alpha subunit V
ITGB3: Integrin-beta subunit 3
M-CSF: Macrophage colony-stimulating factor
MMP9: Matrix metalloproteinase 9
MMP14: Matrix metalloproteinase 14
NFATC1: Nuclear Factor Of Activated T Cells 1
PBMCs: Peripheral blood mononuclear cells
PS: Phosphatidylserine
RANK: Receptor-activator of NF-κB
RANKL: Receptor-activator of NF-κB Ligand
TRAP: Tartrate-resistant acid phosphatase
